# Older adults’ experiences during the COVID-19 pandemic: a qualitative systematic literature review

**DOI:** 10.1186/s12877-023-04282-6

**Published:** 2023-09-20

**Authors:** Elfriede Derrer-Merk, Maria-Fernanda Reyes-Rodriguez, Laura K. Soulsby, Louise Roper, Kate M. Bennett

**Affiliations:** 1 University of Liverpool, Eleanor Rathbone Building, Bedford Street South, Liverpool, L697ZA UK; 2 Universidad de los Andes, Carrera 1 No. 18A-12, Bogotá, Colombia; 3 Principal Health Psychologist, Resilience Hub, Lancashire & South Cumbria Foundation Hospital, Chorley, UK

**Keywords:** Covid-19, Older adults, Risk communication, Social connectivity, Discrimination, Well-being, Coping

## Abstract

**Objectives:**

Relatively little is known about the lived experiences of older adults during the COVID-19 pandemic. We systematically review the international literature to understand the lived experiences of older adult’s experiences during the pandemic.

**Design and methodology:**

This study uses a meta-ethnographical approach to investigate the included studies. The analyses were undertaken with constructivist grounded theory.

**Results:**

Thirty-two studies met the inclusion criteria and only five papers were of low quality. Most, but not all studies, were from the global north. We identified three themes: desired and challenged wellbeing; coping and adaptation; and discrimination and intersectionality.

Overall, the studies’ findings were varied and reflected different times during the pandemic. Studies reported the impact of mass media messaging and its mostly negative impact on older adults. Many studies highlighted the impact of the COVID-19 pandemic on participants' social connectivity and well-being including missing the proximity of loved ones and in consequence experienced an increase in anxiety, feeling of depression, or loneliness. However, many studies reported how participants adapted to the change of lifestyle including new ways of communication, and social distancing. Some studies focused on discrimination and the experiences of sexual and gender minority and ethnic minority participants. Studies found that the pandemic impacted the participants’ well-being including suicidal risk behaviour, friendship loss, and increased mental health issues.

**Conclusion:**

The COVID-19 pandemic disrupted and impacted older adults’ well-being worldwide. Despite the cultural and socio-economic differences many commonalities were found. Studies described the impact of mass media reporting, social connectivity, impact of confinement on well-being, coping, and on discrimination. The authors suggest that these findings need to be acknowledged for future pandemic strategies. Additionally, policy-making processes need to include older adults to address their needs. PROSPERO record [CRD42022331714], (Derrer-Merk et al., Older adults’ lived experiences during the COVID-19 pandemic: a systematic review, 2022).

## Introduction

In March 2020 the World Health Organisation declared a pandemic caused by the virus SARS-CoV2 (COVID-19) [1]. At this time 118,000 cases in 114 countries were identified and 4,291 people had already lost their lives [2]. By July 2022, there were over 5.7 million active cases and over 6.4 million deaths [2]. Despite the effort to combat and eliminate the virus globally, new variants of the virus are still a concern. At the start of the pandemic, little was known about who would be most at risk, but emerging data suggested that both people with underlying health conditions and older people had a higher risk of becoming seriously ill [3]. Thus, countries worldwide imposed health and safety measures aimed at reducing viral transmission and protecting people at higher risk of contracting the virus [4]. These measures included: national lockdowns with different lengths and frequencies; targeted shopping times for older people; hygiene procedures (wearing masks, washing hands regularly, disinfecting hands); restricting or prohibiting social gatherings; working from home, school closure, and home-schooling.

Research suggests that lockdowns and protective measures impacted on people’s lives, and had a particular impact on older people. They were at higher risk from COVID-19, with greater disease severity and higher mortality compared to younger people [5]. Older adults were identified as at higher risk as they are more likely to have pre-existing conditions including heart disease, diabetes, and severe respiratory conditions [5]. Additionally, recent research highlights that COVID-19 and its safety measures led to increased mental health problems, including increased feelings of depression, anxiety, social isolation, and loneliness, potentially cognitive decline [6–22]. Other studies reported the consequences of only age-based protective health measures including self-isolation for people older people (e.g. feeling old, losing out the time with family) [23–30].

Over the past decade, the World Health Organisation (WHO) has recognised the importance of risk communication within public health emergency preparedness and response, especially in the context of epidemics and pandemics. Risk communication is defined as “the real-time exchange of information, advice and opinions between experts or officials, and people who face a threat (hazard) to their survival, health or economic or social well-being” ([31], p5). This includes reporting the risk and health protection measurements through media and governmental bodies. Constructing awareness and building trust in society are essential components of risk communication [32]. In the context of the pandemic, the WHO noted that individual risk perception helped to prompt problem-solving activities (such as wearing face masks, social distancing, and self-isolation). However, the prolonged perception of pandemic-related uncertainty and risk could also lead to heightened feelings of distress and anxiety [31, 33], see also [34–37].

This new and unprecedented disease provided the ground for researchers worldwide to investigate the COVID-19 pandemic. To date (August 2022), approximately 8072 studies have been recorded on the U.S. National Library of Medicine ClinicalTrials.gov [38] and 12002 systematic reviews have been registered at PROSPERO, concerning COVID-19. However, to our knowledge, there is little known about qualitative research as a response to the COVID-19 pandemic and how it impacted older adults’ well-being [39]. In particular, little is known about how older people experienced the pandemic. Thus, our research question considers: How did older adults experience the COVID-19 pandemic worldwide?

We use a qualitative evidence synthesis (QES) recommended by Cochrane Qualitative and Implementation Methods Group to identify peer-reviewed articles [40]. This provides an overview of existing research, identifies potential research gaps, and develops new cumulative knowledge concerning the COVID-19 pandemic and older adults’ experiences. QES is a valuable method for its potential to contribute to research and policy [41]. Flemming and Noyes [40] argue that the evidence synthesis from qualitative research provides a richer interpretation compared to single primary research. They identified an increasing demand for qualitative evidence synthesis from a wide range of “health and social professionals, policymakers, guideline developers and educationalists” (p.1).

## Methodology

A systematic literature review requires a specific approach compared to other reviews. Although there is no consensus on how it is conducted, recent systematic literature reviews have agreed the following reporting criteria are addressed [42, 43]: (a) a research question; (b) reporting database, and search strategy; (c) inclusion and exclusion criteria; (d) reporting selection methods; (e) critically appraisal tools; (f) data analysis and synthesis. We applied these criteria in our study and began by registering the research protocol with Prospero [44].

### Protocol

The study is registered at Prospero [44]. This systematic literature review incorporates qualitative studies concerning older adults’ experiences during the COVID-19 pandemic.

### Search strategy

The primary qualitative articles were identified via a systematic search as per the qualitative-specific SPIDER approach [45]. The SPIDER tool is designed to structure qualitative research questions, focusing less on interventions and more on study design, and ‘samples’ rather than populations, encompassing:

- S-Sample. This includes all articles concerning older adults aged 60 +  [1].
- P-Phenomena of Interest. How did older adults experience the COVID-19 pandemic?
- D-Design. We aim to investigate qualitative studies concerning the experiences of older adults during the COVID-19 pandemic.
- E-Evaluation. The evaluation of studies will be evaluated with the amended Critical Appraisal Skills Programme CASP [46].
- R-Research type Qualitative

### Information source

The following databases were searched: PsychInfo, Medline, CINAHL, Web of Science, Annual Review, Annual Review of Gerontology, and Geriatrics. A hand search was conducted on Google Scholar and additional searches examined the reference lists of the included papers. The keyword search included the following terms: (older adults or elderly) AND (COVID-19 or SARS or pandemic) AND (experiences); (older adults) AND (experience) AND (covid-19) OR (coronavirus); (older adults) AND (experience) AND (covid-19 OR coronavirus) AND (Qualitative). Additional hand search terms included e.g. senior, senior citizen, or old age.

### Inclusion and exclusion criteria

Articles were included when they met the following criteria: primary research using qualitative methods related to the lived experience of older adults aged 60 + (i.e. the experiences of individuals during the COVID-19 pandemic); peer-reviewed journal articles published in English; related to the COVID-19 pandemic; empirical research; published from 2020 till August 2022.

Articles were excluded when: papers discussed health professionals’ experiences; diagnostics; medical studies; interventions; day-care; home care; or carers; experiences with dementia; studies including hospitals; quantitative studies; mixed-method studies; single-case studies; people under the age of 60; grey literature; scoping reviews, and systematic reviews. We excluded clinical/care-related studies as we wanted to explore the everyday experiences of people aged 60 + . Mixed-method studies were excluded as we were interested in what was represented in solely qualitative studies. However, we acknowledge, that mixed-method studies are valuable for future systematic reviews.

### Meta-ethnography

The qualitative synthesis was undertaken by using meta-ethnography. The authors have chosen meta-ethnography over other methodologies as it is an inductive and interpretive synthesis analysis and is uniquely “suited to developing new conceptual models and theories” ([47], p 2), see also [48]. Therefore, it combines well with constructivist grounded theory methodology. Meta-ethnography also examines and identifies areas of disagreements between studies [48].

This is of particular interest as the lived experiences of older adults during the COVID-19 pandemic were likely to be diverse. The method enables the researcher to synthesise the findings (e.g. themes, concepts) from primary studies, acknowledging primary data (quotes) by “using a unique translation synthesis method to transcend the findings of individual study accounts and create higher order” constructs ([47], p. 2). The following seven steps were applied:

1. Getting started (identify area of interest). We were interested in the lived experiences of older adults worldwide.
2. Deciding what was relevant to the initial interest (defining the focus, locating relevant studies, decision to include studies, quality appraisal). We decided on the inclusion and exclusion criteria and an appropriate quality appraisal.
3. Reading the studies. We used the screening process described below (title, abstract, full text)
4. Determining how the studies were related (extracting first-order constructs- participants’ quotes and second-order construct- primary author interpretation, clustering the themes from the studies into new categories (Table 3).
5. Translating the studies into one another (comparing and contrasting the studies, checking commonalities or differences of each article) to organise and develop higher-order constructs by using constant comparison (Table 3). Translating is the process of finding commonalities between studies [48].
6. Synthesising the translation (reciprocal and refutational synthesis, a lines of argument synthesis (interpretation of the relationship between the themes- leads to key themes and constructs of higher order; creating new meaning, Tables 2, 3),
7. Expressing the synthesis (writing up the findings) [47, 48].

### Screening and Study Selection

**Fig. 1:**
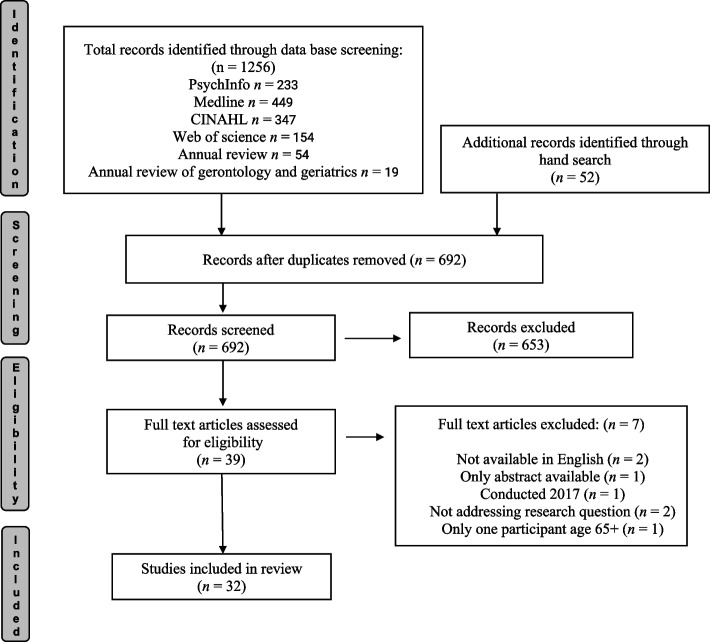
Prisma flow diagram adapted from Page et al. [50]. The PRISMA 2020 statement: an updated guideline for reporting systematic reviews. BMJ, 372, n71. https://doi.org/10.1136/bmj.n71)

A 4-stage screening protocol was followed (Fig. 1 Prisma). First, all selected studies were screened for duplicates, which were deleted. Second, all remaining studies were screened for eligibility, and non-relevant studies were excluded at the preliminary stage. These screening steps were as follows: 1. title screening; 2. abstract screening, by the first and senior authors independently; and 3. full-text screening which was undertaken for almost all papers by the first author. However, 2 papers [9, 23] were assessed independently by LS, LR, and LMM to avoid a conflict of interest. The other co-authors also screened independently a portion of the papers each, to ensure that each paper had two independent screens to determine inclusion in the review [49]. This avoided bias and confirmed the eligibility of the included papers (Fig. 1). Endnote reference management was used to store the articles and aid the screening process.﻿

### Data extraction

After title and abstract screening, 39 papers were selected for reading the full article. 7 papers were excluded after the full-text assessment (1 study was conducted in 2017, but published in 2021; 2 papers were not fully available in English, 2 papers did not address the research question, 1 article was based on a conference abstract only, 1 article had only one participant age 65 +).

The full-text screening included 32 studies. All the included studies, alongside the CASP template, data extraction table, the draft of this article, and translation for synthesising the findings [47, 48] were available and accessible on google drive for all co-authors. All authors discussed the findings in regular meetings.

### Quality appraisal

A critical appraisal tool assesses a study for its trustworthiness, methodological rigor, and biases and ensures “transparency in the assessment of primary research” ([51], p. 5); see also [48–53]. There is currently no gold standard for assessing primary qualitative studies, but different authors agreed that the amended CASPS checklist was appropriate to assess qualitative studies [46, 54]. Thus, we use the amended CASP appraisal tool [42]. The amended CASP appraisal tool aims to improve qualitative evidence synthesis by assessing ontology and epistemology (Table 1 CASP appraisal tool).

**Table 1:** Amended CASP critical appraisal tool [46]

1. Was there a clear statement of the aims?	2. Is a qualitative method appropriate?	3. Was the research design appropriate to address the aims?	4. Was the recruitment strategy appropriate to the aims of the study?	5. Was the data collected in a way that addressed the research issue	6. Has the relationship between researcher and participants been adequately considered?	7. Have ethical issues been taken into account?	8. Was the data analysis sufficiently rigorous?	9. Is there a clear statement of findings?	10. How valuable is the research (High, middle, low)	11. Ontology/epistemology/ Are the study’s theoretical underpinnings clear, consistent and conceptually correct?

A numerical score was assigned to each question to indicate whether the criteria had been met (= 2), partially met (= 1), or not met (= 0) [54]; see also [55]. The score 16 – 22 are considered to be moderate and high-quality studies. The studies scored 15 and below were identified as low-quality papers. Although we focus on higher-quality papers, we did not exclude papers to avoid the exclusion of insightful and meaningful data [42, 48, 52–57]. The quality of the paper was considered in developing the evidence synthesis.

We followed the appraisal questions applied for each included study and answered the criteria either ‘Yes’, ‘Cannot tell’, or ‘No’. (Table 1 CASP appraisal criteria). The tenth question asking the value of the article was answered with ‘high’ of importance, ‘middle’, or low of importance. The new eleventh question in the CASP tool concerning ontology and epistemology was answered with yes, no, or partly (Table 1).

### Data synthesis

The data synthesis followed the seven steps of Meta-Ethnography developed by Noblit & Hare [58], starting the data synthesis at step 3, described in detail by [47]. This encompasses: reading the studies; determining how the studies are related; translating the studies into one another; synthesis the translations; and expressing synthesis. This review provides a synthesis of the findings from studies related to the experiences of older adults during the COVID-19 pandemic. The qualitative analyses are based on constructivist grounded theory [59] to identify the experiences of older adults during the COVID-19 pandemic (non-clinical) populations. The analysis is inductive and iterative, uses constant comparison, and aims to develop a theory. The qualitative synthesis encompasses all text labelled as ‘results’ or ‘findings’ and uses this as raw data. The raw data includes participant’s quotes; thus, the synthesis is grounded in the participant's experience [47, 48, 60, 61]. The initial coding was undertaken for each eligible article line by line. Please see Table 2 Themes per author and country. Focused coding was applied using constant comparison, which is a widely used approach in grounded theory [61]. In particular, common and recurring as well as contradicting concepts within the studies were identified, clustered into categories, and overarching higher order constructs were developed [47, 48, 60] (Tables 2, 3, 4).

**Table 2:** Themes per author and country

Author	Title	Country	Themes of each article	Subthemes	Categories	Higher order constructs
Akkus et al., 2021 [62]	Perceptions and experiences of older people regarding the COVID-19 pandemic process: A phenomenological study	Turkey	The Meaning of COVID-19	Multifaceted Fear, Social Restriction, Biology/Fate Dilemma,	Meaning of Covid-19	Meaning
			COVID-19 Outcomes: Overall Decline in Health and Well-being	Physical, mental, social, and economic outcomes or effects	Health	Wellbeing
			Need for Support and Resources: “We became self-sufficient.”	Economic, health care, informational, and emotional and spiritual support	Support	Wellbeing
			Attitudes toward Vaccination: “Everyone says something different.”	Fear of the vaccine; The Vaccine Offers Hope	Uncertainty	Wellbeing
Banerjee & Rao, 2021 [63]	The Graying Minority": Lived Experiences and Psychosocial Challenges of Older Transgender Adults During the COVID-19 Pandemic in India	India	Marginalization	Second” priority, stigma, social disconnection, perceived loss of dignity	Marginalisation	Wellbeing
			The dual burden of “age” and “gender”	Prominence of ageism	Dual burden	Discrimination- dual burden
				Deprived psychosexual needs, cornered in their communities		
			Multi-faceted survival threats	Psychological, emotional, financial	Health	Wellbeing
			Coping	Social rituals and pride celebrations	Coping	Coping
				Acceptance of the discomfort of beloning to the third gender		
				Spiritulity, hope		Wellbeing
			Unmeet needs	Kowledge, attitude, practice (KAP) related to COVID-19	Unmet needs	
				Social inclusion	Social connectivity	Wellbeing
				Mental health care	Health	Wellbeing
				The audience for their voices	Value	Wellbeing
Brooke, J., & Clark, M., 2020 [64]	Older people’s early experience of household isolation and social distancing during COVID-19	UK/Irland		Handwashing; two-metre social distancing; disinfecting practices; and face masks	Adaptation	Adaptation
			Protective measures	Social media, weather and the garden, tasks to complete	Adaptation	Adaptation
			Current and future plans; and	Blessed, lucky and fortunate; and life still to be lived		
			Acceptance of a good life, but still a life to live		Coping	Coping
Bundy et al., 2021 [65]	The Lived Experience of Already-Lonely Older Adults During COVID-19	USA	Loneliness Did Not Necessarily Compound		Loneliness	Wellbeing
			Managing Loneliness and Enduring Social Isolation		Social isolation	Wellbeing
			Loneliness, Protective, and Responsible		loneliness	Wellbeing
			The Anxieties of COVID-19		Anxiety	Wellbeing
Chemen and Gopalla, 2021 [66]	Lived experiences of older adults living in the community during the COVID-19 lockdown—The case of mauritius	Mauritius	Fears of the virus and fear of deprivation		Health	Wellbeing
			Relieving and recreating bounds		Social connectivity	Wellbeing
			Active contribution to family life		Support	Wellbeing
			Being and feeling valued within the family		Value/family	Wellbeing
			Rediscovering family time and family moments		Value/family	Wellbeing
			Fear of going back to normal		Uncertainty	Adaptation
			Social isolation		Social Isolation	Wellbeing
Derrer-Merk et al., 2022 [9]	Older people's family relationships in disequilibrium during the COVID-19 pandemic. What really matters?	UK	Pre-pandemic to March 2020	Social connectedness	Social connectivity	Wellbeing
				Methods of support	Support	Wellbeing
			Pandemic March to July 2020	Social disconnectedness	Social connectivity	Wellbeing
				Change of desired ad perceived support	support	Wellbeing
Derrer-Merk et al., 2022 [23]	Is protecting older adults from COVID-19 ageism? A comparative cross-cultural constructive grounded theory from the United Kingdom and Colombia	UK/Colombia	Benevolent versus hostile ageism		Ageism	Discrimination
			Society's view on ageing as homogenous		Homogeneous view	Discrimination
			Lost autonomy		Lost autonomy	Wellbeing
			Differences between the UK and Colombia			
Falvo et al., 2021 [67]	Lived experiences of older adults during the first COVID-19 lockdown: A qualitative study	Switzerland	Impact on the individual level: Between fear of going out and a feeling of reclusion	Fear of going out, reclusion	Uncertainty	Wellbeing
			Impact on the micro-social level: The dual role of the other		Social connectivity	Wellbeing
			Impact on the meso-social level: Between protection and stigmatization		Protection/ stigmatization	Wellbeing
			Impact on the macro-social level: Gestation of a new world		New world	Adaptation
Fiocco et al., 2021 [68]	Stress and Adjustment during the COVID-19 Pandemic: A Qualitative Study on the Lived Experience of Canadian Older Adults	Canada	Perceived threat and challenges associated with the pandemic	Threat of contracting the SARA-CoV2 Virus	Threat of contracting the virus	Wellbeing
				Financial Threat	Financial threat	Coping
				Fear Messaging in the Media	Risk communication Risk perception	Wellbeing
				Living Arrangement Challenges		Adaptation
				The Challenge of Physical Distancing and Minimal Social Interactions	Social connectivity	Wellbeing
				The Challenge of Health Management and Health Services	Health management	Wellbeing
				Use of Technology: A New Necessity	Technology	Wellbeing
			Coping with the COVID-19 pandemic	Behavioural strategies	Coping	Coping
				Emotioal-focused strategies	Coping	Coping
				Social support	Support	Wellbeing
Fristedt et al., 2022 [69]	Changes in daily life and wellbeing in adults, 70 years and older, in the beginning of the COVID-19 pandemic	Sweden	Suddenly at risk- … but it could be worse	My world closed down	Threat of the virus	Wellbeing
				Negogiations, adaptaions and prioritazations to manage staying at home	Coping/ adaptation	Adaptation
				Barriers and facilitators to sustain occupational participation	Participation	Wellbeing
				Considerations of my own and other’s health and wellbeing	Health/ wellbeing	Wellbeing
Gazibara et al., 2022 [70]	Experiences and aftermath of the COVID-19 lockdown among community-dwelling older people in Serbia	Serbia	Perception of the curfew announcement;	Being calm	Wellbeing	Wellbeing
				Feeling distressed	Mental health	Wellbeing
				Feeling angry	Distress	
			Attitude towards the curfew;	Positive and compliant		Adaptation
				Negative and resistant	Coping	Coping
			Organization of daily living;	Shopping for groceries	Adaptation	Adaptation
				Access to healthcare services	Health services	Adaptation
				Daytime activities	Adaptation	Adaptation
			Mood		Mood	Wellbeing
			Frustrations/Limitations	Lack of physical activity	Wellbeing	Wellbeing
				Lack of social interactions	Social connectivity	Wellbeing
				Time allocated for walking outside and grocery shopping	Exercise	Wellbeing
			Making sense of the curfew 15 months after		Adaptation	Wellbeing
Giebel et al., 2022 [71]	COVID-19 Public Health Restrictions and Older Adults' Well-being in Uganda: Psychological Impacts and Coping Mechanisms	Uganda	Impact on emotional well-being;	Frustration about situation and boredom	Wellbeing	Wellbeing
				Upset about inability to see friends and family	Social connectivity	Wellbeing
				Fear		
			Implications on physical well-being;	Frailty	Wellbeing	Wellbeing
				Lack of cognitive and social stimulation	Coping	Coping
			Coping mechanisms	Acceptance	Coping	Coping
				Faith	Adaptation	Adaptation
Goins et al., 2021 [72]	Older Adults in the United States and COVID-19: A Qualitative Study of Perceptions, Finances, Coping, and Emotions	USA	Risk Perception	Yes, due to underlying conditions	Risk perception	Wellbeing
				Yes, because of age but with reluctance; yes without reluctance but only because of age; yes with elaboration; no, because they are healthy despite meeting age criteria; no without elaboration		Wellbeing
			Financial impact	Yes negatively; yes positvely; no impact; no, not currently	Impact	Wellbeing
			Coping Problem-focused:	Reduce exposure	Coping	Coping
				Reduce susceptibility	Adaptation	Adaptation
			Emotion-focused:	Creating daily structure	Coping	Coping
				New/creative activities		
				Connecting with others in new ways	Social connectivity	Wellbeing
				Limiting news media exposure	Coping	Wellbeing
			Emotions	Not affected		Coping
				Anxiety, fear, and loneliness	Mental health	Wellbeing
				Disappointments	Frustration	Wellbeing
				Positive feelings	Optimism	Wellbeing
Gomes et al., 2021 [73]	Elderly people's experience facing social isolation in the COVID-19 pandemic	Brazil	Longing for extra-houshold routine and family life		Adaptation	Adaptation
			Building new routine		Coping	Coping
			Fear of the death		Health	Wellbing
			Strategies for preventing COVID-19		Adaptation	Adaptation
			Spirituality and pleasurable activities pre-pandemic		Coping	Coping
			Signs and symptoms experienced during SARS-COV2 infection		Health	Wellbeing
Gonçalves et al., 2022 [74]	Perceptions, feelings, and the routine of older adults during the isolation period caused by the COVID-19 pandemic: a qualitative study in four countries	Brazil, USA,Italy, Portugal	Deprivation	Freedom/right to come and go	Deprivation	Wellbeing
				Restriction from being with others	Social connectivity	Wellbeing
				Changes in leisure	Adaptation	Adaptation
				Restriction of actions of self-management and self-care	Health	Wellbeing
				Work	Purpose in live	Wellbeing
				Medical consultations	Health	Wellbeing
			Adaptation process	Domestic chores	Adaptation	Adaptation
				Recreation/leisure activities	Activities	Wellbeing
				Technological resources	Coping	Coping
				Idleness	Health	Wellbeing
			Coping strategies	Belief, faith, and hope	Coping	Coping
				Information/following recommendations	Health	Wellbeing
				Family	Social connectivity	Wellbeing
			Emotional instability	Negative feelings – Positive feelings	Wellbeing	Wellbeing
			Understanding of COVID-19	Definition	Adaptation	Adaptation
				Transmission – Symptoms	Health	Wellbeing
				Protective measures	Health	Wellbeing
Greenwood-Hickman et al., 2021 [75]	A Qualitative Investigation of Impacts and Coping Strategies During the COVID-19 Pandemic Among Older Adults	USA	General Impacts to Daily Life	Staying at Home	Practical daily impact	Impact
				Travel	Coping	Wellbeing
				Work	Adaptation	Adaptation
				Finances	Coping	Coping
				Policy impacts to behaviour	Adaptation	Adaptation
			Health and Activity Impacts Mental Health, Energy, and Stress	Mental health, engergy, and stress	Health	Wellbeing
				Nutrition	Health	Wellbeing
				Physical Activity	Health	Wellbeing
				Sedentary Time	Health	Wellbeing
				Sleep; sickness/infection with COVID-19	Health	Wellbeing
			Social Impacts Changes to In-person Social Engagement	Family Events	Social impact	Wellbeing
			Coping Strategies			Coping
			Social Connection	Virtual	Social connectivity	Wellbeing
				In person		
			Activities	Hobbies	Adaptation	Adaptation
				Exercise	Health	Wellbeing
				Following Public Health Guidance and Minimizing Risk	Health,	Wellbeing
				HART participation	Participation	Wellbeing
			Beliefs and Attitude	Positive attitude	Adaptation	Adaptation
				Spirituality	Health	Adaptation
Hafford-Letchfieldet al., 2022 [76]	Unheard voices: A qualitative study of LGBT + older people experiences during the first wave of the COVID-19 pandemic in the UK	UK	Risk factors for LGBT + older people and organisations, including specific findings on trans experiences;	Risk factors experienced by LGBT + older people	Risk factors for minorities,Risk perception/experience	Wellbeing
				Specific risks for trans people	Risk factors	Wellbeing
				Risk factors for LGBT + organisations		
			Care practices in LGBT + lives;	Secure relationship/ partnership; offering accommodation to partners; increased visibility of concealed relationships; active outreach to family/ friends; reconnecting/ relationships; fear of formal care/ increase in volunteers; Advocacy in transfer to formal car	Support	Wellbeing
			strengths and benefits of networking	Opportunities to connect with neighbours; Kinder communities; being aware of others needs/ increase take up of services online; role of anonymity	Support social connectivity	Wellbeing
			Politicisation of ageing and their relevance to LGBT + communities	Loss of community advocacy and support; perceived ageism; invisibility; Lack of inclusive services, active outreach to family/friends; effect of rurality on networks/ reduction in campaigning; less visibility in local authorities; lack of information in health and social care; increased fragmentation of services; lack of inclusive services; exclusion from contingency planning; access to additional funding	Impact Support Discrimination Social connectivity	Wellbeing Discrimination
			Learning from communication and provision in a virtual world	Improved virtual services for trans; new peer networks/ increase in volunteers; and take up services online; costs and benefits of adapting services to virtual delivery; new peer networks	Adaptation	Adaptation
Huntley and Bratt, 2022 [77]	An interpretative phenomenological analysis of the lived experiences of older adults during the covid-19 pandemic in Sweden	Sweden	A life on hold	Adherence to restrictions	A life on hold	Wellbeing
				Vaccines—a light at the end of the tunnel	Hope	
			Caring for body and soul, and	Mood	Wellbeing	Wellbeing
				Physical health	Health	Wellbeing
				Everyday meaningfulness	Meaningful live	Wellbeing
			Putting things into perspective	Longing and love	Meaningful live	Wellbeing
				Privilege	Adaptation	Adaptation
				Nostalgia	Reflection	Adaptation
Jiménez-Etxebarria et al., 2021 [78]	Impact of the COVID-19 Pandemic as perceived by Older People in Northern Spain	Spain	Confinement and Perceived Impact on Lifestyle, and Physical and Psychological Health	Activities carried out before the pandemic (volunteering, leisure, exercise, dependent care, learning	Impact health	Wellbeing
				Impact of confinement on activities (interruption of activities, plans we cannot make, excitement due to cessation of activity, I do not know we will be able to return to the activities	Impact of confinement	Wellbeing
				Routine performed in confinement (Description of the routine, adaptive behaviour,	Adaptation	Adaptation
			Health	Impact of confinement on physical condition (I do not see any changes, neagive changes)	Health/wellbeing	Wellbeing
				Impact of confinement in psychological state (same as always, notice changes, negative changes, positive emotions, ambigious or mixed emotions, uncertainty)	Emotional wellbeing	Wellbeing
			Social relationships during confinement	Search for contact maintenance (use of technology,	Social connectivity	Wellbeing
				Contact assessment (satisfaction, this contact cannot be called a relationship, fear of physical contact)	Social connectivity	Wellbeing
				Changes in the form of relationship (contacts we cannot have, new forms of contact, remote family contact)	Social connectivity	Wellbeing
			Older people	Treatment of older people during the confinement (positive perception, negavite perception, nursing homes)	Perception	Wellbeing
			State of confinement or pandemic	Attitudes (percieved negative aspects, notice positive aspects, manifest coping strategies)	Perception / adaptation	Wellbeing
				Reflection on the future (how I value my personal situation, assess the social situation,	Reflection	Wellbeing
Kremers et al., 2022 [79]	The psychosocial adaptability of independently living older adults to COVID-19 related social isolation in the Netherlands	Netherland	‘Social behaviour during the COVID- 19 outbreak’,	Maintenance of contact	Social connectivity	Wellbeing
				Adaptation	Adaptation	Adaptation
				Less contact	Social connectivity	Wellbeing
			‘Emotional behaviour during the COVID- 19 outbreak’			
			Motivation to expand the social network’		Social connectivity	Wellbeing
Kulmala et al., 2021 [80]	Personal Social Networks of Community-Dwelling Oldest Old During the Covid-19 Pandemic	Finland	The Size of the Personal Network Reduced Significantly	Avoiding all places with a lot of people; Fear (own or others) of the virus, Restricting contacts even with the closest family; Meetings outside impossible due to own of other person’s sickness or disability, A relative/friend is a caregiver for someone else and cannot leave home, Hobbies has been closed, Use of digital tools were perceived as difficult and were not applied, Relatives prohibited contacting other people	Social connectivity	Wellbeing
			Personal Networks Remained the Same, but Modifications in Contacting Other People Were Done Based on Recommendations	Phone contacts increased; Relatives, friends and neighbors were met outside and with safety distances; Video, internet and WhatsApp contacts with the family started, Applying safer ways of greeting and meeting people, i.e., not shaking hands anymore, using face masks; Hobbies, i.e., physical activity groups, organized online	Social connectivity	Wellbeing
			Personal Networks Increased During the Pandemic	Spending more time with partner; Contacting friends and relatives who had not been contacted for a long time; More frequent online contacts with children and grandchildren; Feeling socially more connected with the neighbors; Importance of pets increased	Social connectivity	Wellbeing
			Significant or Unexpected Change in Personal Network Happened During the Pandemic	Death of a spouse;Death of a friend; Birth of great grandchildren	Social connectivity	Wellbeing
			The Pandemic Did Not Influence Personal Networks at all	Phone contacts with relatives and friends were as common as previously; Friends and family visited regardless of restrictions or children live close or at the same house; Current personal social network was seen as fulfilling; Enjoying time alone and having no obligations to leave home	Social connectivity	Wellbeing
Mahapatra et al., 2021 [81]	Coping with COVID-19 pandemic: reflections of older couples living alone in urban Odisha, India	Indai	Theme 1: risk appraisal and feeling vulnerable		Risk perception	Wellbeing
			Theme 2: safeguarding against COVID-19		Health	Wellbeing
			Theme 3: managing routine health care and emergency		Adaptation	Adaptation
			Theme 4: pursuing mental and psychological well-being		Wellbeing	Wellbeing
McKinlay et al., 2021 [82]	A qualitative study about the mental health and wellbeing of older adults in the UK during the COVID-19 pandemic	UK	Potential Threats to Wellbeing	Concerns about end-of-life, ageing, and mortality; Thinking about end-of-life concerns, worries about ageing and frailty	Risk perception	Wellbeing
				Grieving the loss of normality; Feeling life is on hold, craving normality, finding the state of the world upsetting	Wellbeing	Wellbeing
				Healthcare concerns;Fear of hospitalisation, fear of seeking help due to perceived lack of service availability, fear of leaving the house due to COVID	Health	Wellbeing
				Unable to engage with activities that protect wellbeing; Loss of leisure, lack of routine	Health	Wellbeing
			Protective Activities and Behaviours	Slowing the pace of life; More time for exercise and new hobbies, time for introspection, and organising affairs	Adaptation	Adaptation
				Benefits of routine and social responsibility; Feeling “needed” and helping others, keeping busy with social obligations	Support / Adaptation	Adaptation
				Social interaction and support; Connecting with others, reciprocal offers of support	Support	Wellbeing
				Utilising skills, experience and resources to cope; Using past coping skills and experience, accustomed to isolation, accessing practical resources	Coping	Coping
Pfabigan et al., 2022 [83]	Under reconstruction: the impact of COVID-19 policies on the lives and support networks of older people living alone.	Austria	Attitudes towards the pandemic situation and its threats	Attitudes	Adaptation	Adaptation
			Dealing with restrictions and hygiene measures		Adaptation	Adaptation
			Managing everyday life and support		Adaptation/ support	Adaptation
			Shifts in support networks			
			Negotiating autonomy		Autonomy	Wellbeing
Prigent et al., 2022 [84]	Intergenerational tension or cohesion during the covid-19 pandemic?: A letter-writing study with older new zealanders	New Zealand	Familial intergenerational interaction		Social connectivity	Wellbeing
			Neighborhood interactions		Social connectivity	Wellbeing
			Societal interactions			
Sangrar et al., 2021 [85]	Exploring the Interpretation of COVID-19 Messaging on Older Adults' Experiences of Vulnerability	Canada	Theme 1: “Fact-Checking”: How Older Adults Interpret Early Information and Factors that Influence their Interpretation	a systematic approach to consuming COVID-19 messaging	Perception of news	Wellbeing
				intrinsic factors of discourse consumption and interpretation	Risk perception	Wellbeing
				Extrinsic factors of discourse interpretation		
			Theme 2: “Just be Careful”: Manifestations of Vulnerability	Emotional responses to early messaging	Risk perception	Wellbeing
				Personalizing pandemic messaging	Risk perception	Wellbeing
			Theme 3: “Changed the Lifestyle”: Impacts of COVID-19 Messaging on Everyday Living	Disrupted Routines	Impact of news risk perception	Wellbeing
				Community engagement	Social connectivity	Wellbeing
			Theme 4: “NotBadin myLocale”: Contextual Considerations for Discourse Interpretation	Pandemic narrative	Impact of news	Wellbeing
				Micro and macro contexts	Impact of news	Wellbeing
Sattari and Billore, 2020 [86]	Bring it on Covid-19: being an older person in developing countries during a pandemic. *Working with Older People*	India/Iran	Perception of risk and fear		Risk perception	Wellbeing
			Change in lifestyle adaptation to the pandemic situation		Adaptation	Adaptation
Verhage et al., 2021 [87]	Coping of Older Adults in Times of COVID-19: Considerations of Temporality Among Dutch Older Adults	Netherland	Situating the Crisis: Meaning in Life		Meaning	Wellbeing
			Coping Strategies During the Crisis	Self-enhancing comparisons (problem focused, emotional focused, meaning focused)	Coping	Coping
				Gaining control by following measures	Adaptation	Adaptation
				Distraction	Adaptation	Adaptation
				Temporary acceptance	Adaptation	Adaptation
				Interpreting individual vulnerability	Risk perception	Risk perception
Wang et al., 2021 [88]	Identities: experiences and impacts of the COVID-19 pandemic from the perspectives of older Chinese immigrants in Canada	Canada	Immigration		Minority experience	Discrimination
			Older age		Self perception	Wellbeing
			Racism towards people of Chinese descent?		Racism	Discrimination
			Family Roles as older parents and grandparents		Role	Identity
			Use of technology		Technology	Wellbeing
Xie et al., 2021 [89]	Living Through the COVID-19 Pandemic: Community-Dwelling Older Adults’ Experiences	USA	Theme 1: positive experiences	Perception that the pandemic had not changed ones lifestyle	Impact on lifestyle	Wellbeing
				Adjusting well—particularly with the aid of thechnology	Adaptation	Adaptation
				Being positive in perspective		
				The loner advantage	Loner advantage	Wellbeing
			Theme 2: mixed experiences	Doing well but unhappy about having to change lifestyle routines	Adaptation	Adaptation
				Doing well but unhappy about not having in-person interactions	Social connectivity	Wellbeing
				Doing well but frustrated by others' behaviors		
				Maintaining physical health with fluctuations of isolation and symptoms of depression or anxiety	Health Social connectivity	Wellbeing
			Theme 3: negative experiences	Bitter about others( e.g. society, government) not caring for older adults	Impact Neglect	Wellbeing
				Feeling isolated, bored, and powerless	Impact	Wellbeing
				Worsening as time goes by		
Yang et al., 2021 [90]	The Experiences of Community-dwelling older adults during the COVID-19 Lockdown in Wuhan	China	Challenges posed by COVID- 19	Tight medical resources	Limited resources Health	Wellbeing
				Inconvenience in daily life	Inconvenience	Adaptation
				Negative emotions	Wellbeing	Wellbeing
			Support during the COVID- 19 epidemic	Social support	Support	Wellbeing
				Technical support		
			Resilience amid challenges	coping in daily lives	Coping	Coping
				Transcendence		
			Impact after the COVID- 19 epidemic	Mental burdens	Impact	Wellbeing
				Sense of benefit from the lockdown	Benefit	Wellbeing
Yıldırım, H., 2022 [91]	Psychosocial status of older adults aged 65 years and over during lockdown in Turkey and their perspectives on the outbreak	Turkey	Growing old is like a crime	Finding comfort	Wellbeing	Wellbeing
			The inevitable course		Adaptation	Wellbeing
			The cost of lockdown at home	Coping well	Coping	Coping
			The desire for equality	Equality	Equality	Discrimination

**Table 3:** From codes to categories and higher order constructs

**Higher order constructs**	**Desired and challenged wellbeing** [3, 9, 23, 62, 63, 65–70, 72, 75–85, 88–90]	**Coping and adaptation** [63, 64, 68, 69, 72, 75, 79, 81, 85, 87–90]	**Discrimination – intersectionality (age and race/ gender identity** [23, 62, 63, 67, 70, 76, 84, 88]
Categories	Risk perception- communication Social connectivity Impact of confinement on well-being	Emotional Behavioural	Ageism Racism Heterosexism
Code examples	Threat of the virus Financial threat Ageing and mortality Media, news perception Health Physical distancing Support- family- friends Personal networks Value Social isolation loneliness Unmet needs Physical activity Mood change	Hygiene routines Use of technology New activities Change of daily routines- adhering to restrictions- slowing the pace Positive attitude Spirituality Hope -vaccine	Marginalisation Risk factors Dual burden Homogeneous view Lost autonomy Loss of community Lack of services Immigration Racism Ageism Gender discrimination Equality

**Table 4:** Characteristics of studies and participants and presence of analytical themes. (^a^Higher order constructs: Risk perception and risk communication; coping and adaptation; Discrimination- intersectionality)

Author	Research aims	Country	Recruitment strategy	Participants’ age	Number of participants	Methodology	CASP score	Higher order constructs^a^		
								1	2	3
Akkus et al., 2021 [62]	To examine thoroughly the perceptions and experiences of older people regarding the COVID-19 outbreak	Turkey	Purposeful snowball sampling	10 women, 6 men, age range 65 -80	16	Content analysis	18	X		
Banerjee & Rao, 2021 [63]	To explore the lived experiences and psychosocial challenges of older transgender adults during the COVID-19 pandemic in India	India	Purposeful snowball sampling from LGBTQ community, Index participant was known	Age range 64- 71, mean age 66.4	10	Hasse’s adaptation of Colaizzi’s phenomenological	19	X	X	X
Brooke & Clark, 2020 [64]	To explore older people's initial experience of household isolation, social distancing and shielding, and the plans they constructed to support them through the COVID-19 pandemic	UK, Rep Ireland	Snowball and mouth to word	Age range 70—89 mean age 77 (5.77 SD) 4 Cabirian, 1 European, 13 English	19	Inductive phenomenology	21		X	
Bundy et al., 2021 [65]	To understand how already-lonely older individuals navigated and endured the social isolation of the pandemic	USA	Conducted with patients of a large health care system during assessment	65 + + age range 65–92 average 73, 7 women, 5 men	12	Constant comparative method	21	X		
Chemen & Gopalla, 2021 [66]	To explored the lived experiences of older adults living in the community during the COVID-19 sanitary lockdown in the small island state of Mauritius	Mauritius	Convenience snowball sampling	3 men, 12 women, mean age 69.6 (SD 6.88) no age range available	15	Thematic analysis	22	X		
Derrer-Merk et al., 2022b [9]	To explores how older people in the United Kingdom experienced changes in inter- and intragenerational support during the COVID-19 pandemic	UK	COVID-19 psychological research consortium	65 + age range 65- 83 mean 71, SD 5; 18 women, 15 men, 18 living alone 15 not	33	Constructivist grounded theory	21	X		
Derrer-Merk, et al., 2022c [23]	To explore consequence of COVID-19 measures established new form of ageism in the United Kingdom and Colombia	UK/Colombia	COVID-19 psychological research consortium and snowball sampling	65/60; UK 65 + age range 65- 83 mean 71, SD 5; 18 women, 15 men, 18 living alone 15 not? CO 32 age range 63–95, mean 69 SD 9, 16 men, 16 women, each 8 living alone or not	65	Constructivist grounded theory	21	X		X
Falvo et al., 2021 [67]	To explore the lived experiences of individuals aged 64 or older during the first COVID-19 lockdown	Ticino/ Switzerland	Available database from the local source population	64 + age range 64–85, 12 women, 7 men, average age 75, SD 6,04	19	Inductive thematic analysis	19	X		
Fiocco et al., 2021 [68]	To understand the lived experience of community dwelling older adults during the first six months of the pandemic in Ontario, Canada	Canada	Snowball sampling within Stress and Healthy Aging Research Lab and community partners	65 + , age range 65–81, 13 women, 9 men, average 72.33, SD 4.25,	22	Inductive thematic analysis	18	X	X	
Fristedt et al., 2022 [69]	To explore how adults 70 + experienced and managed changes in everyday life due to the COVID-19 pandemic and how those changes affected wellbeing at the beginning of the virus outbreak	Sweden	Part of the ‘At Risk Study’, a qualitative longitudinal project	70 + 11 women, 6 men, mean age 76, age range 71–87	17	Qualitative context analysis	18	X	X	
Gazibara et al., 2022 [70]	To examine the experiences and perceptions of curfew for older people in Serbia 15 months after the curfew had ended	Serbia	Snowball sampling	65 + , 15 women, 8 men, age range 66–90, mean age 72.4, SD 6.2	23	Descriptive information, using naturalistic theoretical orientation. Qualitative content analysis	18	X	X	
Giebel et al., 2022 [71]	To explore the psychological effects of COVID-19 public health measures on older adults in Uganda and their coping mechanisms	Uganda	Purpose sampling, Snowball sampling	60 + , 23 women, 7 men, no other information	30	Deductive thematic analysis	14	X	X	
Goins et al., 2021 [72]	To understand COVID-related perceptions and behaviours of older adults residing in the United States	USA	Master students recruited each 2 participants, convenience sampling	65 + mean age 72.4 SD 6.7 age range 65–92, 24 women, 19 men	43	Low-inference qualitative descriptive design	18	X	X	
Gomes et al., 2021 [73]	To unveil the experience of the elderly with social isolation in the pandemic of COVID-19	Brazil	Not mentioned	60 + age range 60–79	14	Inductive classification of words IRAMUTEC and multivariate analysis	14		X	
Gonçalves et al., 2022 [74]	To evaluate the knowledge, routine, and perception of older adults from four countries about dealing with COVID-19 in the social isolation period	Brazil, United States, Italy, and Portugal	Snowball technique	Mean age varied from 65.8 to 72.4 Brazil 69.5 (SD 6.2), USA 68.4 (SD 10.6), Portugal 72.4 (SD 7.6), Italy 65.8 (SD 3.7) total male 6, women 19	25	Content analysis based on thematic units	13	X	X	
Greenwood-Hickman et al., 2021 [75]	To explore the physical, mental, and social health impacts of the pandemic on older adults and their coping techniques	USA	HART randomized controlled trial'- recruited from Kaiser Permanente Washington membership panels in King County, WA	16 women, 8 men, 1 non-binary, mean age 68, range 60–77 no SD	25	Inductive thematic approach assisted by Atlas	20	X	X	
Hafford-Letchfield et al., 2022 [76]	To report immediate impact of social distancing measures on the lives (LGBT +)	UK	Nothing mentioned	60 + age from the professional is not known, 60–74, LGBT, 12 women, 5 men	17 LGBT older adults + 6 professionals from the LGBT community centre	Content analysis, from audio and memos, no transcripts	16	X	X	X
Huntley & Bratt, 2022 [77]	To explore the lived experiences of eight older adults in Sweden, of living during a pandemic	Sweden	Convenience sampling snowball sampling	70 + age range 71–82 four men, four women	8	Interpretative phenomenological analysis (IPA) using diaries across a 14-day period, followed by interviews	21	X		
Jiménez-Etxebarria et al., 2021 [78]	To explore the perspective, perception, attitudes, treatment, and changes of people over 67	Spain	Convenience sampling, snowball technique	67 age range 68–81, 6 men, 20 women, (no mean or SD)	26	Inductive approach	21	X	X	
Kremers et al., 2022 [79]	To explore independently living older adults’ perceptions of social and emotional well- being during the COVID- 19- related self- isolation, and their motivation to expand their social network in the future	Netherland	Snowball sampling Local newspaper and website advertisement, ‘Netwerk 100’, and the personal network of the researchers	56 + age range 56–87, mean age 72, (SD 7.5) 11 women, 9 men	20	Open coding process, grounded theory approach	19	X	X	
Kulmala et al., 2021 [80]	To investigated changes in personal networks among community-dwelling oldest-old individuals (persons aged 80 and over) during the first and second waves of the COVID-19 pandemic in Finland	Finland	Cardiovascular Risk Factors, Aging, and Dementia (CAIDE85 +) study	80 + age mean, 84.8 Sd 7.3, 10 women, 5 men,	15	Directed content analyses	16	X		
Mahapatra, et al., 2021 [81]	To explore the ‘coping reflections’ of elderly couples living alone (without any other family members) during the COVID-19 pandemic in urban Odisha, India	India	Our study was nested within a larger community-based study	65 + couples living alone, 11 couples = 22 participants	11	Interpretive thematic analysis	20	X	X	
McKinlay et al., 2021 [82]	To examine factors that threatened and protected the wellbeing of older adults living in the UK during social distancing restrictions due to the COVID-19 pandemic	UK	Purposive, snowball sampling	70 + 9 women, 11 men, average 79 age range 70 s -90 s	20	Reflexive thematic analysis	21	X	X	
Pfabigan et al., 2022 [83]	To explore how the COVID-19 containment policies affected older people living alone	Austria	The sub-study is part of the OPLA study	Age range79-94 mean 85; 1 mn, 6 women	7	Framework method	20	X	X	
Prigent et al., 2022 [84]	To explore experiences of intergenerational interaction during the first COVID-19 lockdown in Aotearoa, New Zealand (NZ)	New Zealand	Nationwide Snowball sampling	70 + / 13 younger as s70 age range 60–94, 67% women, 29 men, 3 no gender	412 letters	Reflexive thematic analysis	20	X		
Sangrar et al., 2021 [85]	To examined perspectives on COVID-19 messaging	Canada	Purposive sampling	65 + 67- 91 age range, average 75.4, SD 7.0, 14 women, 4 men	18	Inductive thematic analysis	20	X		
Sattari & Billore, 2020 [86]	To explore the respective risk perception toward the Covid-19 pandemic among the elderly in two developing countries	India and Iran	Not described	60–85, age range 65–83, 12 men, 10 women,	22	Not described	9	X	X	
Verhage et al., 2021 [87]	To explore how Dutch older adult’s view this crisis and cope with measures	Netherland	Snowball sampling	54–95. age range 54–95, mean age 75.5, 34 women, 25 men	59	Constant comparison	22	X	X	
Wang et al., 2021 [88]	To understand the unique experiences of older Chinese adults in Canada in the early stages of the COVID-19 pandemic	Canada	Criterion sampling, purposive sampling	65 + , age range 65–83, average 73, 8 women, 7 men	15	Thematic analysis	21	X		X
Xie et al., 2021 [89]	To address the gap of COVID-19 pandemic’s impact of community-dwelling older adults’ lived experiences during this historical period	USA, Texas	Snowball sampling through local organizations (e.g., senior centers, Meals on Wheels)	65–92. mean age 73.6, SD 6.33, 138 women 62 men,	200	Inductive thematic analysis	17	X	X	
Yang et al., 2021 [90]	To explore the experiences of community- dwelling older adults in Wuhan during the coronavirus disease 2019 lockdown	China	Purposive and snowball sampling	65 + women 10, men 8, mean age 72, SD 5.53	18	Colaizzi's phenomenological approach	19	X	X	
Yildirim, 2022 [91]	To identify the psychosocial status, attitudes, and experiences of individuals aged 65 and over who were in- home lockdown during the COVID- 19 outbreak in Turkey	Turkey	Snowball sampling method	65 + , mean age 71.33 SD 5.26, age range 65–91, women 23, men 28	51	Thematic analysis	13	X	X	X

^a^Theme 1: Risk perception and risk communication

Theme 2: Coping and adaptation

Theme 3: Discrimination- intersectionality

## Findings

We identified twenty-seven out of thirty-two studies as moderate-high quality; they met most of the criteria (scoring 16/22 or above on the CASP; [54]. Only five papers were identified as low qualitative papers scoring 15 and below [71, 73, 74, 86, 91]. Please see the scores provided for each paper in Table 4. The low-quality papers did not provide sufficient details regarding the researcher’s relationship with the participants, sampling and recruitment, data collection, rigor in the analysis, or epistemological or ontological reasoning. For example, Yildirim [91] used verbatim notes as data without recording or transcribing them. This article described the analytical process briefly but was missing a discussion of the applied reflexivity of using verbatim notes and its limitations [92].

This systematic review found that many studies did not mention the relationship between the authors and the participant. The CASP critical appraisal tool asks: Has the relationship between the researcher and participants been adequately considered? (reflecting on own role, potential bias). Many studies reported that the recruitment was drawn from larger studies and that the qualitative study was a sub-study. Others reported that participants contacted the researcher after advertising the study. One study Goins et al., [72] reported that students recruited family members, but did not discuss how this potential bias impacted the results.

**Fig. 2:**
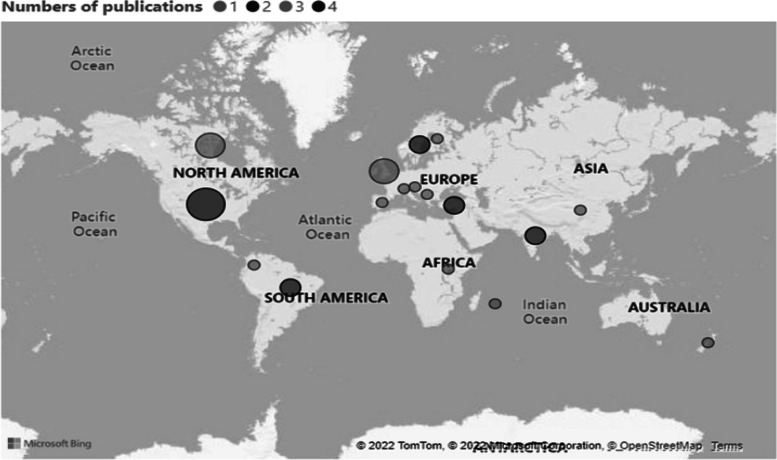
Numbers of publications by country

Our review brings new insights into older adults’ experiences during the pandemic worldwide. The studies were conducted on almost all continents. The majority of the articles were written in Europe followed by North America and Canada (4: USA; 3: Canada, UK; 2: Brazil, India, Netherlands, Sweden, Turkey 2; 1: Austria, China, Finland, India/Iran, Mauritius, New Zealand, Serbia, Spain, Switzerland, Uganda, UK/Ireland, UK/Colombia) (see Fig. 2). Note, as the review focuses on English language publications, we are unable to comment on qualitative research conducted in other languages see [72].

The characteristics of the included studies and the presence of analytical themes can be found in Table 4. We used the following characteristics: Author and year of publication, research aims, the country conducted, Participant’s age, number of participants, analytical methodology, CASP score, and themes.

We identified three themes: desired and challenged wellbeing; coping and adaptation; discrimination and intersectionality. We will discuss the themes in turn.

### Desired and challenged wellbeing

Most of the studies reported the impact of the COVID-19 pandemic on the well-being of older adults. Factors which influenced wellbeing included: risk communication and risk perception; social connectivity; confinement (at home); and means of coping and adapting. In this context, well-being refers to the evidence reported about participants' physical and mental health, and social connectivity.

#### Risk perception and risk communication

Politicians and media transmitted messages about the response to the pandemic to the public worldwide. These included mortality and morbidity reports, and details of health and safety regulations like social distancing, shielding- self-isolation, or wearing masks [34–37]. As this risk communication is crucial to combat the spread of the virus, it is also important to understand how people perceived the reporting during the pandemic.

Seven studies reported on how the mass media impacted participants' well-being [23, 67, 68, 70, 72, 81, 85]. Sangrar et al. [68] investigated how older adults responded to COVID-19 messaging: “My reaction was to try to make sure that I listen to everything and [I] made sure I was aware of all the suggestions and the precautions that were being expressed by various agencies …”. (p. 4). Other studies reported the negative impact on participants' well-being of constant messaging and as a consequence stopped watching the news to maintain emotional well-being [3, 67, 68, 70, 72, 81, 85]. Derrer-Merk et al. [23] reported one participant said that “At first, watching the news every day is depressing and getting more and more depressing by the day, so I’ve had to stop watching it for my own peace of mind” (p. 13). In addition, news reporting impacted participants’ risk perception. For example, “Sometimes we are scared to hear the huge coverage of COVID-19 news, in particular the repeated message ‘older is risky’, although the message is useful.” ([81], p5).

#### Social connectivity

Social connectivity and support from family and community were found in fourteen of the studies as important themes [9, 62, 66–68, 75–80, 83, 84, 90].

The impact of COVID-19 on social networks highlighted the diverse experiences of participants. Some participants reported that the size of social contact was reduced: “We have been quite isolated during this corona time” ?([80], p. 3). Whilst other participants reported that the network was stable except that the method of contact was different: “These friends and relatives, they visited and called as often as before, but of course, we needed to use the telephone when it was not possible to meet” ([77], p. 5). Many participants in this study did not want to expand their social network see also [9, 77–79]. Hafford-Letchfield et al. [76] reported that established social networks and relationships were beneficial for the participants: “Covid has affected our relationship (with partner), we spend some really positive close time together and support each other a lot” (p. 7).

On the other hand, other studies reported decreases of, and gaps in, social connectedness: “I couldn’t do a lot of things that I’ve been doing for years. That was playing competitive badminton three times a week, I couldn’t do that. I couldn’t get up early and go volunteer in Seattle” [9, 67, 75]. A loss of social connection with children and grandchildren was often mentioned: “We cannot see our grandchildren up close and personal because, well because they [the parents] don’t want us, they don’t want to risk our being with the kids … it’s been an emotional loss exacerbated by the COVID thing” ([68] p.10); see also [9, 67, 78]. On the contrary, Chemen & Gopalla [66] note that those older adults who were living with other family members reported that they were more valued: “Last night my daughter-in-law thanked me for helping with my granddaughter” (p.4).

Despite reports of social disconnectedness, some studies highlighted the importance of support from family members and how support changed during the COVID-19 pandemic [9, 62, 81, 83, 90]. Yang et al. [90] argued that social support was essential during the Lockdown in China: “N6 said: ‘I asked my son-in-law to take me to the hospital” (p. 4810). Mahapatra et al. [81] found, in an Indian study, that the complex interplay of support on different levels (individual, family, and community) helped participants to adapt to the new situation. For example, this participant reported that: “The local police are very helpful. When I rang them for something and asked them to find out about it, they responded immediately” (p. 5).

#### Impact of confinement on well being

Most articles highlighted the impact of confinement on older adults’ well-being [9, 62, 63, 65, 67, 69, 70, 72, 75, 77–79, 81–83, 85, 89, 90].

Some studies found that participants maintained emotional well-being during the pandemic and it did not change their lifestyle [79, 80, 82, 83, 89, 92]: “Actually, I used this crisis period to clean my house. Bookcases are completely cleaned and I discarded old books. Well, we have actually been very busy with those kind of jobs. So, we were not bored at all” ([79], p. 5). In McKinlay et al. [82]’s study, nearly half of the participants found that having a sense of purpose helped to maintain their well-being: “You have to have a purpose you see. I think mental resilience is all about having a sense of purpose” (p. 6).

However, at the same time, the majority of the articles (12 out of 18) highlighted the negative impact of confinement and social distancing. Participants talked of increased depressive feelings and anxiety. For example, one of Akkus et al.’s [62] participants said: “... I am depressed; people died. Terrible disease does not give up, it always kills, I am afraid of it …” (p. 549). Similarly, one of Falvo et al.’s [67] participants remarked: “I am locked inside my house and I am afraid to go out” (p. 7).

Many of the studies reported the negative impact of loneliness as a result of confinement on participants’ well-being including [69, 70, 72, 78, 79, 90, 93]. Falvo et al. [67] reported that many participants experienced loneliness: “What sense does it make when you are not even able to see a family member? I mean, it is the saddest thing not to have the comfort of having your family next to you, to be really alone” (p. 8).

Not all studies found a negative impact on loneliness. For example, a “loner advantage” was found by Xie et al. ([82], p. 386). In this study participants found benefits in already being alone “It’s just a part of who I am, and I think that helps—if you can be alone, it really is an asset when you have to be alone” ([82], p. 386).

Bundy et al. [80] investigated loneliness from already lonely older adults and found that many participants did not attribute the loneliness to the pandemic: “It’s not been a whole lot, because I was already sitting around the house a whole lot anyway ( …). It’s basically the same, pretty well … I’d pretty well be like this anyway with COVID or without COVID” (p. 873) (see also [83]).

A study from Serbia investigated how the curfew was perceived 15 months afterward. Some participants were calm: “I realized that … well … it was simply necessary. For that reason, we accepted it as a measure that is for the common good” ([70], p.634). Others were shocked: “Above all, it was a huge surprise and sort of a shock, a complete shock because I have never, ever seen it in my life and I felt horrible, because I thought that something even worse is coming, that I even could not fathom” ([70], p. 634).

The lockdowns brought not only mental health issues to the fore but impacted the physical health of participants. Some reported they were fearful of the COVID-19 pandemic: “... For a little while I was afraid to leave, to go outside. I didn’t know if you got it from the air” ([75]. p. 6). Another study reported: “It’s been important for me to walk heartily so that I get a bit sweaty and that I breathe properly so that I fill my lungs—so that I can be prepared—and be as strong as possible, in case I should catch that coronavirus” ([77], p. 9); see also [70, 78, 82, 85].

### Coping and adaptation

Many studies mentioned older adults’ processes of coping and adaptation during the pandemic [63, 64, 68, 69, 72, 75, 79, 81, 85, 87–90].

A variety of coping processes were reported including: acceptance; behavioural adaptation; emotional regulation; creating new routines; or using new technology. Kremers et al. [79] reported: “We are very realistic about the situation and we all have to go through it. Better days will come” (p. e71). Behavioural adaptation was reported: “Because I’m asthmatic, I was wearing the disposable masks, I really had trouble breathing. But I was determined to find a mask I could wear” ([68], p. 14). New routines with protective hygiene helped some participants at the beginning of the pandemic to cope with the health threat: “I am washing my hands all the time, my hands are raw from washing them all the time, I don't think I need to wash them as much as I do but I do it just in case, I don’t have anybody coming in, so there is nobody contaminating me, but I keep washing” ([69], p. 4391); see also [72]. Verhage et al. [87] reported strategies of coping including self-enhancing comparisons, distraction, and temporary acceptance: “There are so many people in worse circumstances …” (p. e294). Other studies reported how participants used a new technology: “I have recently learned to use WhatsApp, where I can make video phone calls.” ([88], p. 163); see also [89].

### Discrimination -intersectionality (age and race/gender identity)

Seven studies reported ageism, racism, and gender discrimination experienced by older adults during the pandemic [23, 63, 67, 70, 76, 84, 88].

Prigent et al. [84], conducted in a New Zealand study, found that ageism was reciprocal. Younger people spoke against older adults: “why don’t you do everyone a favour and drop dead you f******g b**** it’s all because of ones like you that people are losing jobs” (p. 11). On the other hand, older adults spoke against the younger generation: “Shame to see the much younger generations often flout the rules and generally risk the gains made by the team. Sheer arrogance on their part and no sanctions applied” (p.11). Although one study reported benevolent ageism [23] most studies found hostile ageism [23, 63, 67, 70, 76, 84]. One study from Canada exploring 15 older adult’s Chinese immigrants’ experiences reported racism as people around them thought they would bring the virus into the country. The negative impact on existing friendships was told by a Chinese man aged 69 “I can tell some people are blatantly despising us. I can feel it. When I talked with my Caucasian friends verbally, they would indirectly blame us for the problem. Eventually, many of our friendships ended because of this issue” ([88], p161). In addition, this study reported ageism when participants in nursing homes felt neglected by the Canadian government.

Two papers reported experiences of sexual and gender minorities (SGM) (e.g. transgender, queer, lesbian or gay) and found additional burdens during the pandemic [63, 76]. People experienced marginalisation, stereotypes, and discrimination, as well as financial crisis: “I have faced this throughout life. Now people look at me in a way as if I am responsible for the virus.” ([63], p. 6). The consequence of marginalisation and ignorance of people with different gender identities was also noted by Hafford- Letchfield et al. [76]: “People have been moved out of their accommodation into hotels with people they don't know …. a gay man committed suicide, community members know of several that have attempted suicide. They are feeling pretty marginalised and vulnerable and you see what people are writing on the chat pages” (p.4). The intersection of ageism, racism, and heterosexism and its negative impact on people’s well-being during the pandemic reflects additional burden and stressors for older adults.

## Discussion

This systematic literature review is important as it provides new insights into the lived experiences of older adults during the COVID-19 pandemic, worldwide. Our study highlights that the COVID-19 pandemic brought an increase in English-written qualitative articles to the fore. We found that 32 articles met the inclusion criteria but 5 were low quality. A lack of transparency reduces the trustworthiness of the study for the reader and the scientific community. This is particularly relevant as qualitative research is often criticised for its bias or lack of rigor [94]. However, their findings are additional evidence for our study.

Our aim was to explore, in a systematic literature review, the lived experiences of older adults during the COVID-19 pandemic worldwide. The evidence highlights the themes of desired and challenged wellbeing, coping and adaptation, and discrimination and intersectionality, on wellbeing.

Perceived risk communication was experienced by many participants as overwhelming and anxiety-provoking. This finding supports Anwar et al.’s [37] study from the beginning of the pandemic which found, in addition to circulating information, that mass media influenced the public's behaviour and in consequence the spread of disease. The impact can be positive but has also been revealed to be negative as well. They suggest evaluating the role of the mass media in relation to what and how it has been conveyed and perceived. The disrupted social connectivity found in our review supports earlier studies that reported the negative impact of people’s well-being [6–28] at the beginning of the pandemic. This finding is important for future health crisis management, as the protective health measures such as confinement or self-isolation had a negative impact on many of the participants’ emotional wellbeing including increased anxiety, feelings of depression, and loneliness during the lockdowns. As a result of our review, future protective health measures should support people’s desire to maintain proximity with their loved ones and friends. However, we want to stress that our findings are mixed.

The ability of older adults to adapt and cope with the health crisis is important: many of the reported studies noted the diverse strategies used by older people to adapt to new circumstances. These included learning new technologies or changing daily routines. Politicians and the media and politicians should recognise both older adults' risk of disease and its consequences, but also their adaptability in the face of fast-changing health measures. This analysis supports studies conducted over the past decades on lifespan development, which found that people learn and adapt livelong to changing circumstances [95–97].

We found that discrimination against age, race, and gender identity was reported in some studies, in particular exploring participants’ experiences with immigration backgrounds and sexual and gender minorities. These studies highlighted the intersection of age and gender or race and were additional stressors for older adults and support the findings from Ramirez et al. [98] This review suggests that more research should be conducted to investigate the experiences of minority groups to develop relevant policies for future health crises.

Our review was undertaken two years after the pandemic started. At the cut-off point of our search strategy, no longitudinal studies had been found. However, in December 2022 a longitudinal study conducted in the USA explored older adult’s advice given to others [99]. They found that fostering and maintaining well-being, having a positive life perspective, and being connected to others were coping strategies during the pandemic [100]. This study supports the results of the higher order constructs of coping and adaptation in this study. Thus, more longitudinal studies are needed to enhance our understanding of the long-term consequences of the COVID-19 pandemic. The impact of the COVID-19 restrictions on older adults’ lives is evident. We suggest that future strategies and policies, which aim to protect older adults, should not only focus on the physical health threat but also acknowledge older adults' needs including psychological support, social connectedness, and instrumental support. The policies regarding older adult’s protections changed quickly but little is known about older adults’ involvement in decision making [100]. We suggest including older adults as consultants in policymaking decisions to ensure that their own self-determinism and independence are taken into consideration.

There are some limitations to this study. It did not include the lived experiences of older adults in care facilities or hospitals. The studies were undertaken during the COVID-19 pandemic and therefore data collection was not generally undertaken face-to-face. Thus, many studies included participants who had access to a phone, internet, or email, others could not be contacted. Additionally, we did not include published papers after August 2022. Even after capturing the most commonly used terms and performing additional hand searches, the search terms used might not be comprehensive. The authors found the quality of the papers to be variable, and their credibility was in question. We acknowledge that more qualitative studies might have been published in other languages than English and were not considered in this analysis.

To conclude, this systematic literature review found many similarities in the experiences of older adults during the Covid-19 pandemic despite cultural and socio-economic differences. However, we stress to acknowledge the heterogeneity of the experiences. This study highlights that the interplay of mass media reports of the COVID-19 pandemic and the policies to protect older adults had a direct impact on older adults’ well-being. The intersection of ‘isms’ (ageism, racism, and heterosexism) brought an additional burden for some older adults [98]. These results and knowledge about the drawbacks of health-protecting measures need to be included in future policies to maintain older adults’ well-being during a health crisis.

## Data Availability

The systematic literature review is based on already published articles. And all data analysed during this study are included in this manuscript. No additional data was used.
